# Suggestions on the Preparation of the Lying-in Chamber

**Published:** 1903-08

**Authors:** W. B. M’Laughlin

**Affiliations:** Oklahoma City, O. T.


For Texas Medical Journal.

### Suggestions on the Preparation of the Lying-in Chamber.

BY W. B. M'LAUGHLIN, M. D., OKLAHOMA CITY, O. T.

In all the domain of science there is no prouder achievement than Oliver Wendell Holmes's proof of the contagiousness of puerperal fever. In 1861 the capstone was set upon this discovery by Summelweis, who demonstrated that the hands of the obstetrician usually carried the infection, and devised a method of hand disinfection.

While much has been done in working out the details of the life history of the bacteria causing this infection, we have advanced but little from the period where these pioneers laid down the work, when we consider that the whole science of aseptic and antiseptic surgery has had its birth since Summelweis penned his classical essay. The labor of many brilliant minds has brought surgery through the antiseptic period up to the aseptic period, and today the surgeon is almost as careful about the room in which he operates as he is of the field upon which he operates. But it seems that the obstetrician has slept through all this period of wonderful development. He disinfects his hands as Summelweis did forty years ago, and calmly sits down, feeling that his whole duty is done. And if now and then a case of puerperal fever or *phlegmasia alba dolens* develops in his practice, he says it was unavoidable, and serenely goes to sleep again. Why has the obstetrician confined himself to disinfection of his hands and instruments and paid so little attention to the preparation of the lying-in chamber?

It is true that the surgeon can prepare a special room for operating, and keep it in good condition, while the obstetrician is called upon to do his work in all sorts of conditions. Until recently no means of disinfection has been available which would thoroughly disinfect a room without serious damage to its contents, but now sanitarians have given us formaldehyde gas and several enterprising American companies have developed generators to such a degree of perfection that there now remains no excuse for the obstetrician to lag behind his brother surgeon in the preparation of his operating room.

For the practical application of this idea I hope I may take the liberty of making the following suggestions: Three or four days before the expected confinement, the room in which the patient expects to spend the puerperal period should be thoroughly cleaned, the windows closed, and all cracks stopped up with wax paper. A

clothes line is then stretched several times across the room, and on this line is hung the bedding which is to be used during the period, as well as the gowns, lochial cloths, etc., of the patient. The room is now filled with formaldehyde gas (2 to 2.5 per cent per volume of air in the room) and closed. It is allowed to remain closed for twenty-four hours, when, if the moist gas has been used, it will be found necessary to open and air the room thoroughly. It is then closed until the day of confinement. If, however, the dry gas has been used, the room need not be opened until just before the time when it is to be occupied by the woman, if a little ammonia spray be used to neutralize the formaldehyde which has polymerized into pariform. Therefore, in my practice, I prefer the dry gas. It is claimed that moist formaldehyde is a more effective germicide, but bacteriological tests have shown that the dry gas in 2 per cent solution in air will destroy sporulating anthrax through two thicknesses of billiard cloth. I, therefore, regard the dry gas as eminently preferable and sufficient for practical sterilization.

It is my opinion that by a thorough room disinfection on the above suggested principles, in conjunction with methods now in use, puerperal sepsis will soon be a matter simply of historical interest.

